# Relating hepatocellular carcinoma tumor samples and cell lines using gene expression data in translational research

**DOI:** 10.1186/1755-8794-8-S2-S5

**Published:** 2015-05-29

**Authors:** Bin Chen, Marina Sirota, Hua Fan-Minogue, Dexter Hadley, Atul J Butte

**Affiliations:** 1 Division of Systems Medicine, Department of Pediatrics, Stanford University School of Medicine, 1265 Welch Road MSOB X163, Stanford, CA, 94305-5415, USA

## Abstract

Cancer cell lines are used extensively to study cancer biology and to test hypotheses in translational research. The relevance of cell lines is dependent on how closely they resemble the tumors being studied. Relating tumors and cell lines, and recognizing their similarities and differences are thus very important for translational research. Rapid advances in genomics have led to the generation of large volumes of genomic and transcriptomic data for a diverse set of primary cancer samples, normal tissue samples and cancer cell lines. Hepatocellular Carcinoma (HCC) is one of the most common tumors worldwide, with high occurrence in Asia and sub-Saharan regions. The current effective treatments of HCC remain limited. In this work, we compared the gene expression measurements of 200 HCC tumor samples from The Cancer Genome Atlas and over 1000 cancer cell lines including 25 HCC cancer cell lines from Cancer Cell Line Encyclopedia. We showed that the HCC tumor samples correlate closely with HCC cell lines in comparison to cell lines derived from other tumor types. We further demonstrated that the most commonly used HCC cell lines resemble HCC tumors, while we identified nearly half of the cell lines that do not resemble primary tumors. Interestingly, a substantial number of genes that are critical for disease development or drug response are either expressed at low levels or absent among highly correlated cell lines; additional attention should be paid to these genes in translational research. Our study will be used to guide the selection of HCC cell lines and pinpoint the specific genes that are differentially expressed in either tumors or cell lines.

## Introduction

Cancer cell lines are widely used to study cancer biology and to test hypotheses in translational research [1,2]. Numerous cancer cell lines are derived from tumors, and are used for various purposes such as understanding disease progression, developing diagnostics, and screening anti-tumor drugs. However, the relevance of a cell line is dependent on how closely it resembles the tumors being studied, while the *in vitro *cell culture environment differs from that of *in vivo *tumor tissue. Moreover, not all cancer cell lines have equal value or relevance as tumor models [3]. Therefore, relating tumors and cell lines, and recognizing their similarities and differences is of critical importance in translational research and understanding cancer biology.

It has been shown that genetic and epigenetic changes including gene mutations, deletions, amplifications, translocations and methylation status found in lung tumors are retained in lung cancer cell lines [4]. Prolonged cell culture is more likely to cause the secondary genomic changes such as copy number variation and gene expression [3]. Several previous studies have compared the genomic and transcriptomic differences between tumors and cell lines, but their sample size has been very limited [5-8]. Integrating disease tissue gene expression and drug gene expression profiled in cancer cell lines for therapeutic discovery has been extensively applied by our lab and others [9-13]. Rapid advances in the field of genomics have led to the generation of a high volume of molecular data across various tumors and cell lines. Of particular note, The Cancer Genome Atlas (TCGA) Research Network has characterized the genomic and gene expression profiles of over 10,000 human tissue samples cross 32 tumor types [14]. The Cancer Cell Line Encyclopedia (CCLE) provides the genomic and gene expression profiles of over 1000 cell lines [15]. Domcke *et al*. recently evaluated ovarian cell lines as tumor models by comparing their genomic profiles published in CCLE and TCGA, and they found that some rarely used cell lines might be more appropriate to study ovarian cancers [16]. To our knowledge, here we present the first comprehensive comparison of tumor and cell line samples using gene expression data specifically for liver cancer.

Liver cancer is the sixth most frequent cancer globally with higher occurrences in Asia and sub-Saharan regions and is the second leading cause of global cancer deaths. The incidence and mortality of liver cancer have increased in the United States and Europe in the past decade. It was projected to become the third leading cause of cancer-related death by 2030 in the USA [17]. While HCC is the most common type of liver cancer, the current effective treatments of HCC remain limited. HCC tumors are insensitive to conventional chemotherapy, although limited benefits have been shown from the targeted drug sorafenib on only a small subset of tumors [18]. Meanwhile, a large number of liver cell lines have been developed and used to screen compounds, and many drugs are showing promising *in vitro *models. However, that these best drug candidates fail to be effective clinically [19] indicates the necessity to examine the correlation between HCC tumors and cancer cell lines and elucidate their similarities and differences for more effective translation of bench-side discovery into clinical utility.

In this work, we compared 200 HCC tumor samples from TCGA to over 1000 cancer cell lines from CCLE using their gene expression profiles, and we found that most HCC cell lines are significantly correlated to the primary tumors in comparison to other cancer cell lines. However, from the set of 25 HCC cell lines, nearly half are not significantly correlated to the tumors. We also found that a small subset of the tumors is not significantly correlated to HCC cell lines. We further identified the differentially expressed genes between the tumor samples and cell lines, and we found that a substantial number of differentially expressed genes are located in the extra cellular space. The genes that are over-expressed in the tumor samples are related to immune response, drug metabolism and ABC transporter. This work helps guide us in optimal selection of cell lines and highlights the differentially expressed genes that play an essential role in translating from *in vitro *to *in vivo *and clinical research.

## Results

### Correlation of gene expression between HCC tumor samples and cancer cell lines

**Figure 1:**
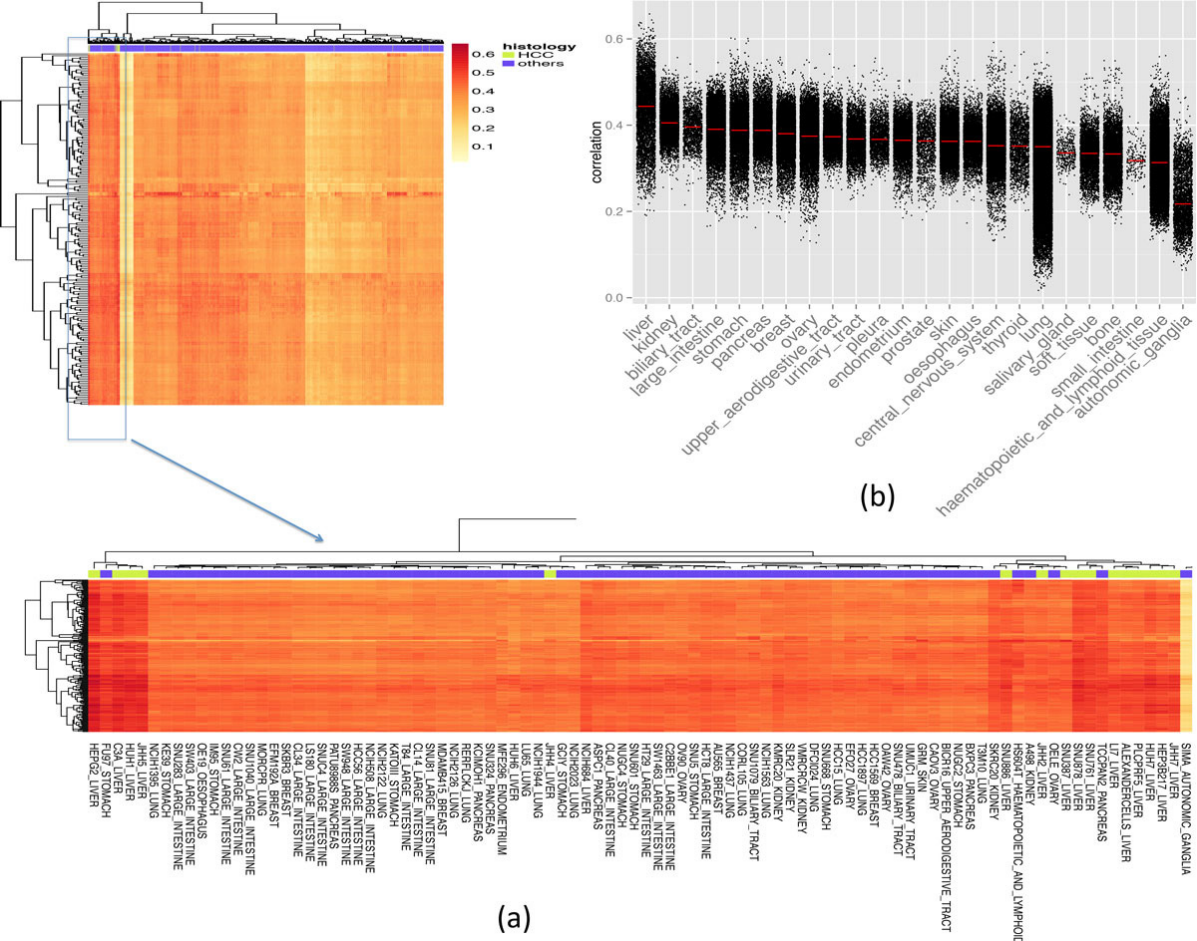
**Spearman correlation between HCC tumors and all cancer cell lines (a) individual pairs (b) grouped by cell line primary site**. In the heatmap, each row represents one tumor sample; each column represents one cell line. The correlation level is represented by color: higher correlation shown in red, lower in yellow. The HCC cell lines are labeled in green. In the boxplot, each dot represents the correlation between one tumor sample and one cell line and the red line is the median.

We compared 200 HCC tumors profiled by RNASeq from TCGA with 1019 cancer cell lines profiled by microarray from CCLE using the top 5000 varying genes (see Methods). There are 28 cancer cell lines derived from liver tissues and 25 of them are related to HCC. Fourteen HCC related cell lines are significantly correlated to the tumors, and 7 non-liver cancer cell lines are correlated (P value < 0.05, Figure 1a). Of particular note, pancreatic cell line TCC-PAN2 (median correlation coefficient of 0.49, P value = 8.8E-3) and stomach cell line FU97 (median correlation coefficient of 0.50, P value = 5.5E-3) are even more correlated with the HCC tumors than some of the HCC cell lines. This suggests that quite a few cell lines originated from another site share similar gene expression profiles with HCC tumors, and they may be used to inform the study of HCC as well.

Among 24 cell line groupings differentiated by their tissue of origin, we found liver cancer cell lines are most correlated to the HCC tumors (median correlation coefficient of 0.44, Figure 1b). The overall correlation is significantly higher than the second and third most correlated cell types, which are the kidney and biliary tract cancer cell lines (median correlation coefficient of 0.41, P value < 1E-16; 0.40, P value < 1E-16, respectively). The three least correlated cell line types are autonomic ganglia (median correlation coefficient of 0.22), hematopoietic and lymphoid tissue (median correlation coefficient of 0.31) and small intestine (median correlation coefficient of 0.32). According to TCGA tissue sample requirements, all tumors were taken at the initial site of cancer. Thus we conclude that HCC cell lines retain the characteristics of the liver origins.

### Correlation of gene expression between HCC tumors and HCC cell lines

However, when looking closely at the distribution of the correlations between HCC tumors and HCC cell lines, we notice that the variation is rather high, with value ranging from 0.19 to 0.66, and standard deviation 0.07 (Figure 1b).

**Figure 2:**
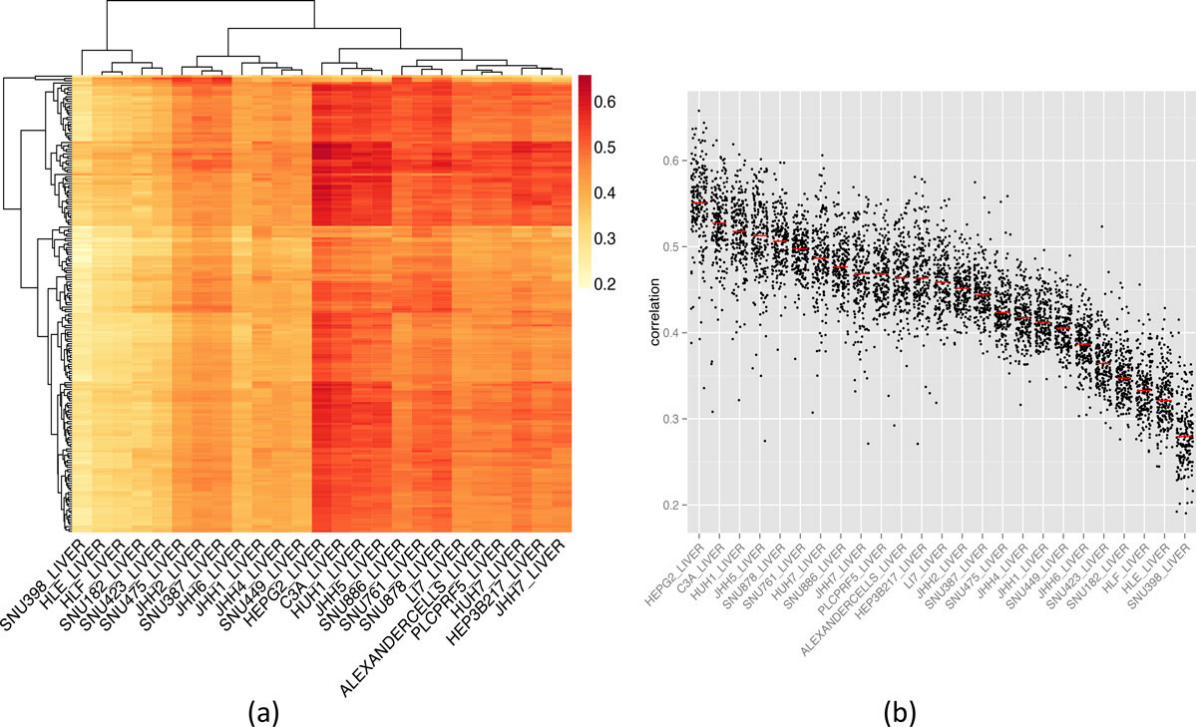
**Spearman correlation between HCC tumors and HCC cancer cell lines (a) individual pairs (b) grouped by cell line**. In the heatmap, each row represents a tumor sample; each column represents one HCC cell line. The correlation level is represented by color: higher correlation shown in red, lower in yellow. In the boxplot, each dot represents the correlation between one tumor sample and one cell line and the red line is the median.

Moreover, we observe that there are two groups of cell lines and one group is more correlated with the tumor samples than the other on the hierarchical clustering of the individual cell lines (Figure 2a). The four most commonly used cell lines (i.e., HepG2, HuH 7, Hep3B and PLC/PRF/5) [19] have significantly high correlations with the tumor samples (P value < 0.05). Interestingly, HepG2, one of the most widely used cell line, has the highest correlation (median correlation coefficient of 0.55, P value = 3.4E-4). However, 11 out of 25 cell lines are not significantly correlated to the tumor samples by comparing to other cell lines (P value > 0.05).

Table 1 lists all HCC cell lines and the medians of their correlation coefficients across the 200 HCC tumors, ranked by their correlation coefficient value. Although we could not find any factors that could be associated with the correlations due to the small sample size as well as incomplete information about some cell lines, several observations are noteworthy. The cell lines sharing high SNP identity (e.g., HepG2 & C3A, PLC/PRF/5 & Alexander cells, and HLF & HLE) tend to have similar correlations. We find that for the SNU series, all the most recently established cell lines (i.e., SNU-878, SNU-761, and SNU-886) are highly correlated but none of the older ones are significantly correlated. The publication of the newer established cell lines did not report the explicit difference between the two versions of cell lines [20]. The cells that are not well differentiated in HLF and HLE may account for their low correlations with the tumors.

**Table 1:** HCC cell lines and their correlations with tumors.

Cell Line	Correlation (P value)	Sex	Ethnicity/Race	Age	Known cancer etiology or other characteristics
HepG2	0.55 (3.4E-4)	M	Caucasian/NA	15	No evidence of a Hepatitis B virus genome

C3A	0.53 (1.1E-3)	M	Caucasian/NA	15	No evidence of a Hepatitis B virus genome; derivative of HepG2

huH-1	0.52 (1.9E-3)	M	Asian/Japanese	53	HBs-antigen carrier

JHH-5	0.51 (3.3E-3)	M	Asian/Japanese	50	Small liver cancer observed in the chronic hepatitis; integration of the HBV-DNA was not observed

SNU-878	0.51 (3.3E-3)	F	Asian/Korean	54	One of newer cell lines in the SNU series

SNU-761	0.5 (5.5E-3)	M	Asian/Korean	49	One of newer cell lines in the SNU series

HuH-7	0.49 (8.8E-3)	M	Asian/Japanese	57	Well differentiated hepatocellular carcinoma

SNU-886	0.48 (1.4E-2)	M	Asian/Korean	57	One of newer cell lines in the SNU series

JHH-7	0.47 (2.1E-2)	M	Asian/Japanese	53	HBs-Ag positive hepatocellular carcinoma with liver cirrhosis

PLC/PRF/5	0.47 (2.1E-2)	M	NA	24	Malignant liver cancer with HBsAg positive

Alexander cells	0.46 (3.1E-2)	M	NA	24	Malignant liver cancer with HBsAg positive

Hep 3B2.1-7	0.46 (3.1E-2)	M	Black/NA	8	Hepatitis B virus DNA was detected

Li-7	0.46 (3.1E-2)	NA	NA	NA	NA

JHH-2	0.45 (4.5E-2)	M	Asian/Japanese	57	Integration of the HBV-DNA was not observed

SNU-387	0.44 (0.06)	F	Asian/Korean	41	Patient treated by transcatheter arterial embolization with lipoidol plus a combination of doxorubicin and mitomycin-C

SNU-475	0.42 (0.12)	M	Asian/Korean	43	Taken from a patient prior to cytotoxic therapy

JHH-4	0.42 (0.12)	M	Asian/Japanese	51	HBs antigen-negative; HCV was not detected

JHH-1	0.41 (0.16)	M	Asian/Japanese	50	Complication of hepatic cirrhosis and hepatocellular carcinoma

SNU-449	0.41 (0.16)	M	Asian/Korean	52	Hepatitis B virus DNA was detected; taken from a patient prior to cytotoxic therapy

JHH-6	0.39 (0.25)	F	Asian/Japanese	57	HBV-DNA was not integrated; undifferentiated hepatocellular carcinoma

SNU-423	0.36 (0.44)	M	Asian/Korean	40	Hepatitis B virus DNA was detected; treated by transcatheter arterial embolization with lipoidol plus doxorubicin

SNU-182	0.35 (0.51)	M	Asian/Korean	24	Taken from a patient prior to cytotoxic therapy

HLF	0.33 (0.64)	M	NA	68	Hepatoma, non-differentiated

HLE	0.32 (0.70)	M	NA	68	Hepatoma, non-differentiated

SNU-398	0.28 (0.89)	M	Asian/Korean	42	Hepatitis B virus (HBV) DNA was detected; treated by transcatheter arterial embolization with lipoidol plus a combination of doxorubicin and mitomycin-C

Median of correlation coefficients is used.

**Figure 3:**
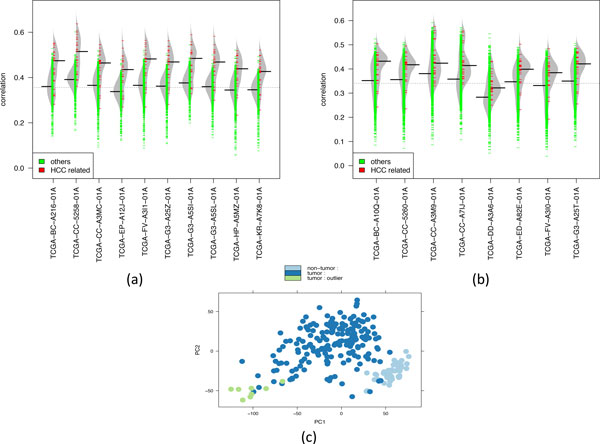
**Example tumor samples significantly/poorly correlated to the HCC cell lines**. (a) Ten tumor samples significantly correlated with the HCC cell lines. (b) Outlier tumor samples poorly correlated with the HCC cell lines. (c) Principal Component Analysis of the tumors (dark blue and green) and non-tumor samples (light blue) from TCGA, the outlier samples in (b) are shown in green.

Additionally, we find that the majority of the tumor samples are individually significantly correlated to the HCC cell lines (a few samples are exampled in Figure 3a). We identify 8 tumor samples as outliers (Figure 3b), after using tissue samples randomly selected from the Expression Project for Oncology [21] to compute the false discovery rate (FDR ≤ 0.05, see Methods). Interestingly, these 8 tumors are similar with each other and are different from the majority of tumors according to the principal component analysis of their gene expression profiles (Figure 3c). This suggests that the current cell lines may be incapable of modeling this subset of tumors based on gene expression analysis. In addition, based on the analysis of clinical features of the outlier samples (Table 2) we are not able to identify any clinical features that are significantly associated with this subset of tumors due to the small sample size.

**Table 2:** Eight tumors poorly correlated to HCC cell lines.

Sample ID	Gender	Race	Tumor status	Vital status	Grade	Residual	Metastasis stage	Pathologic tumor stage
TCGA-BC-A10Q-01A	Female	White	With Tumor	Dead	NA	R1	MX	NA

TCGA-CC-5260-01A	Female	Asian	Tumor Free	Alive	G1	RX	M0	Stage IIIC

TCGA-G3-A25T-01A	Female	White	Tumor Free	Alive	G2	R0	M0	Stage IIIA

TCGA-CC-A3M9-01A	Male	Asian	Tumor Free	Alive	G3	R0	M0	Stage IIIA

TCGA-FV-A3I0-01A	Female	White	Tumor Free	Alive	G2	R0	M0	Stage II

TCGA-DD-A3A6-01A	Female	White	Tumor Free	Dead	G2	R0	M0	Stage II

TCGA-CC-A7IJ-01A	NA	NA	NA	NA	NA	NA	NA	NA

TCGA-ED-A82E-01A	Female	Asian	Tumor Free	Alive	G2	R0	M0	Stage IIIA

Fisher test (P value)	0.01	0.69	0.68	0.76	0.16	0.45	1	0.16

Two-tailed Fisher's exact test was used for associating clinical features to the tumors.

### Difference of gene expression between HCC tumors and HCC cell lines

Most HCC tumors and cell lines share remarkably similar gene expression profiles. However, since the environment of cells growing *in vitro *is different from that in a heterogeneous tissue, the prolonged process of cell culture may induce substantial changes of the expression of some genes. Furthermore drug response *in vitro *is different from that *in vivo *or in the clinic. For example, HCC tumors are often insensitive to some chemotherapy drugs while HCC cell lines are sensitive to those drugs [22]. Therefore, we hypothesize that differentially expressed genes between tumors and cell lines may play important roles in disease development or drug response.

In this regard, we first rank the commonly measured genes between HCC tumor samples and HCC cell line samples based on their expression values in individual samples. For each gene, we computed the difference of the ranks between tumor samples and cell lines to identify differentially expressed genes. Among 17,386 commonly measured genes, 385 are over expressed in tumors compared to cell lines and 279 are over expressed in cell lines compared to tumors (P values < 0.01, log 2 fold change > 2, data in the additional file 1: Table S1-2). We then ask if these genes are relevant to disease development. We reason that the differentially expressed genes between tumors and non-tumors are related to the disease development. We find 1003 genes being significantly differentially expressed between the tumor samples and non-tumor samples from TCGA. A significant number of over expressed genes in tumors compared to cell lines are dysregulated in tumors compared to non-tumors (30 common genes, P value = 0.008, hypergeometric test, data in the additional file 1: Table S3-5). This suggests that the over expressed genes in tumors compared to cell lines are indeed important for the disease development.

We further perform GO and KEGG pathway enrichment analysis of the differentially expressed genes between tumor samples and cell lines using DAVID [23]. For those genes over expressed in tumors compared to cell lines, the top three enriched cellular components are extracellular region, extracellular space and extracellular region part, the top three enriched biological processes are immune response, response to wounding and defense response, and the top three enriched pathways are complement and coagulation cascades, drug metabolism and ABC transporters (Table 3). For those genes over expressed in cell lines compared to tumors, the top three enriched cellular components are intermediate filament, intermediate filament cytoskeleton, and extracellular region, the top three enriched biological processes are G-protein coupled receptor protein signaling pathway, cell surface receptor linked signal transduction and sensory perception of smell, and the top one enriched pathway is Olfactory transduction (Table 4).

**Table 3:** Enriched GO terms and KEGG pathways for over expressed genes in tumors

	Term	P Value	Benjamini (<0.1)
Cellular Component	GO:0005576~extracellular region	8.85E-11	2.22E-08
	
	GO:0005615~extracellular space	2.87E-08	3.60E-06
	
	GO:0044421~extracellular region part	5.49E-08	4.59E-06
	
	GO:0031226~intrinsic to plasma membrane	3.24E-05	2.03E-03
	
	GO:0005887~integral to plasma membrane	4.03E-05	2.02E-03
	
	GO:0005886~plasma membrane	5.35E-04	2.21E-02
	
	GO:0005626~insoluble fraction	6.52E-04	2.31E-02
	
	GO:0005624~membrane fraction	7.56E-04	2.35E-02
	
	GO:0044459~plasma membrane part	1.44E-03	3.94E-02
	
	GO:0000267~cell fraction	1.62E-03	3.98E-02
	
	GO:0005792~microsome	2.90E-03	6.42E-02
	
	GO:0042598~vesicular fraction	3.65E-03	7.36E-02

Biological Process	GO:0006955~immune response	4.00E-11	6.31E-08
	
	GO:0009611~response to wounding	9.12E-10	7.19E-07
	
	GO:0006952~defense response	3.32E-09	1.75E-06
	
	GO:0002253~activation of immune response	2.74E-07	1.08E-04
	
	GO:0006954~inflammatory response	4.09E-07	1.29E-04
	
	GO:0050778~positive regulation of immune response	9.19E-07	2.42E-04
	
	GO:0006956~complement activation	1.26E-06	2.83E-04
	
	GO:0002541~activation of plasma proteins involved in acute inflammatory response	1.52E-06	2.99E-04
	
	GO:0006959~humoral immune response	2.98E-06	5.22E-04
	
	GO:0006958~complement activation, classical pathway	1.70E-05	2.68E-03
	
	GO:0002684~positive regulation of immune system process	1.77E-05	2.54E-03
	
	GO:0002526~acute inflammatory response	2.09E-05	2.75E-03
	
	GO:0002455~humoral immune response mediated by circulating immunoglobulin	2.55E-05	3.09E-03
	
	GO:0048584~positive regulation of response to stimulus	6.24E-05	7.00E-03
	
	GO:0045087~innate immune response	8.20E-05	8.58E-03

KEGG pathway	hsa04610:Complement and coagulation cascades	1.40E-07	1.66E-05
	
	hsa00982:Drug metabolism	1.78E-03	9.99E-02
	
	hsa02010:ABC transporters	2.12E-03	8.02E-02

**Table 4:** Enriched GO terms and KEGG pathways for over expressed genes in cell lines

	Term	P Value	Benjamini (<0.1)
Cellular Component	GO:0005882~intermediate filament	4.05E-09	6.65E-07
	
	GO:0045111~intermediate filament cytoskeleton	5.54E-09	4.54E-07
	
	GO:0005576~extracellular region	2.00E-08	1.10E-06
	
	GO:0045095~keratin filament	3.11E-06	1.27E-04

Biological Process	GO:0007186~G-protein coupled receptor protein signaling pathway	7.66E-11	8.79E-08
	
	GO:0007166~cell surface receptor linked signal transduction	1.82E-07	1.04E-04
	
	GO:0007608~sensory perception of smell	4.78E-07	1.83E-04
	
	GO:0007606~sensory perception of chemical stimulus	5.94E-07	1.70E-04
	
	GO:0050877~neurological system process	7.86E-07	1.80E-04
	
	GO:0007600~sensory perception	9.35E-07	1.79E-04
	
	GO:0050890~cognition	8.63E-06	1.41E-03
	
	GO:0042742~defense response to bacterium	5.24E-04	7.24E-02

KEGG pathway	hsa04740:Olfactory transduction	4.46E-09	2.14E-07

We find that the differentially expressed genes are primarily located in the extracellular space. Moreover, the genes related to immune systems are over expressed in tumors; an observation was previously reported [8]. Interestingly, many genes related to drug metabolism are also over expressed in HCC tumors as well. Liver is the primary organ for drug metabolism, whereas, the cell lines fail to express some of these genes. For example, CYP2C8, the primary enzyme for drug Paclitaxel, is expressed very low across all the HCC cell lines. Paclitaxel has very good sensitivity *in vitro *(with median IC50 100 nm, accessed in ChEMBL) but has no significant anti-tumor effect clinically [24]. The difference of its metabolism profiles between cell lines and tumors may shed light on different drug responses. The expression and activity of drug metabolism enzymes are demonstrated to be extremely low in HCC cell lines, particularly in HepG2 [25-28]. Although we see that the cell lines retain the expression of most genes, the differentially expressed genes between tumors and cell lines are very important to investigate while we translate our discovery from *in vitro *to *in vivo*.

## Discussion

An increasing number of HCC cell lines have been developed for the study of cancer biology and translational research [29], however, only a few cell lines are often chosen for *in vitro *or *in vivo *studies due to limited experimental capability. Choosing appropriate cell lines for experimental testing is thus of critical importance. Unfortunately, crucial information about cell lines (e.g., metastasis status, hepatitis virus status, drug treatment, mutation) is often missing or ambiguous in cell line databases and relevant publications, which further confounds the selection process. As the genomic and transcriptomic profiles of cancer cell lines and tumors become more publically available, using gene expression to relate cancer cell lines to tumor samples becomes a more useful tool to inform cell line selection and has been applied to other cancers (e.g., ovarian cancer [16]). Although gene expression is not the only factor, it can be used to exclude certain cell lines that do not share similar profiles with the tumors being studied.

Our gene expression analysis of tumors and cell lines demonstrates that HCC cell lines are very tissue specific. They resemble the majority of HCC tumors using top 5000 varying genes. This conclusion still holds if top 1000 varying genes are used (data not shown). The four widely used cell lines (i.e., HepG2, HuH 7, Hep3B and PLC/PRF/5) are highly correlated to the tumors. However, nearly half of the cell lines are strikingly not significantly correlated to the tumors. Poor differentiation or genomic damage by chemotherapy drugs may account for the poor correlations of some cell lines.

Our analysis also shows that there is substantial variation of individual tumor samples in regards to their correlations with cell lines. Intrinsic heterogeneity among samples may account for the variation. Specifically, we identify 8 out of 200 tumor samples as outliers that have poor correlations with the known HCC cell lines. It may suggest that the drug tested in these cell lines will most likely have a different clinical effect in the outlier patients. It is interesting to further associate these tumors to clinical features so that this subset of tumors can be easily identified clinically and personalized treatments can be designed. However, the current 8 samples are insufficient to draw a robust conclusion. It may be also interesting to associate them to other genetic features.

Even though many HCC cell lines have overall high correlations with HCC tumors, a large number of genes are actually absent or lowly expressed in cell lines. A significant number of these genes are involved in disease development. Some genes are related to immune system and pharmacokinetics including drug metabolism and drug transportation. It's known that immune system and pharmacokinetics are related to drug response [30]. Inducing missing or low expressed genes in cell lines by external agents may be a way to improve cell line models [26,31].

In summary, by leveraging publicly available transcriptomic data, we relate individual HCC tumors and cancer cell lines. We demonstrate that the most commonly used HCC cell lines closely resemble HCC tumors based on their gene expression, and we also identify nearly half of HCC cell lines do not resemble HCC tumors. Even in the highly correlated cell lines, a significant number of genes that are critical for disease development or drug response are absent or expressed at low levels, therefore, additional attention should be paid to these genes in translational research. Our future work includes covering more tumor samples from pubic sources (e.g., Gene Expression Omnibus) and more cell lines (the Sanger Cancer Cell Line project [32]), analyzing genetic features (e.g., mutation, copy number variation, single nucleotide polymorphism), and linking the subtype of patients classified by clinical features or molecular features to cancer cell lines. Finally, this work serves as a model analysis that can be extended to other tumor types of interest.

## Methods

### Datasets

We downloaded the cell line gene expression file (CCLE_Expression_Entrez_2012-09-29.gct) and the annotation file (CCLE_sample_info_file_2012-10-18.txt) from the CCLE website (http://www.broadinstitute.org/ccle). Gene symbols in the expression file were converted into GeneIDs using AILUN [33]. In total, we analyzed 1019 cell lines with gene expression and annotation data. We collected relevant data of the cell lines including age, sex, race and other characteristics from ATCC (http://www.atcc.org), HSRRB (http://cellbank.nibio.go.jp/english/), and KCLB (http://cellbank.snu.ac.kr).

The level 3 released gene expressions for RNASeqV2 of HCC were downloaded from GDAC (http://gdac.broadinstitute.org). The RSEM abundance estimation processed by the TCGA workgroup was used in the following analysis. The sample clinical data was downloaded from TCGA (https://tcga-data.nci.nih.gov/tcga/). In total, we analyzed HCC 200 tumor samples and 50 non-tumor samples. According to TCGA, tumors samples are the primary tumors, namely the tumors at the initial site of cancer. Normal tissue samples are matched to the anatomic site of the tumor but usually not matched to the participant. Without any notes, tumor samples are specifically referred to HCC. We also downloaded all the samples in expO (GSE2109) from Gene Expression Omnibus.

### Correlation between tumor samples and cell lines

The top 5000 genes ranked by interquartile range across all cell lines were chosen. Among them, 136 genes that are not matched to any genes in tumor samples were ignored in the following analysis. Since the expressions of tumor samples and cell lines were derived from two different technologies and were transformed via different methods [34], we used the ranked-based spearman correlation to assess the similarity between cell lines and tumor samples. For each cell line, the median of its correlations with all tumors was computed. The medians of all cancer cell lines were normally distributed. According to the distribution of the correlations, the significance of the correlation of each cell line with tumor samples was computed (P value < 0.05).

For each tumor sample, Mann-Whitney test was used to test the difference of its correlations with HCC cell lines and with all other cell lines. Random 1000 tissue samples from different origins were taken from expO and further used to correct the P value from the Mann-Whitney test. Only the tumors with P value better than 95% of the random tissue samples were considered as being significantly correlated to the HCC cell lines.

### Differentially expressed genes between tumors and cell lines

The common genes between tumor samples and cell lines samples were ranked based on their expression in individual samples. T-test was used to assess the difference of the ranks between tumor samples and cell lines for individual genes and P values were adjusted by the Benjamini-Hochberg for multiple hypothesis testing. Fold change was computed as the ratio between the mean of ranks across tumor samples and the mean of ranks across cell lines. Log2 fold change > 2 and q value <= 0.01 were used to select differentially expressed genes.

### Differentially expressed genes between tumors and non-tumors

DESeq [35] was used to normalize RNA-Seq data and compute differentially expressed genes. Log2 fold change > 2 and q value <= 0.01 were used to select differentially expressed genes.

### Software tools

All the analysis was conducted in R programming language. Bioconductor packages Beanplot, ggplot2 and pheatmap were used for visualization. GO and KEGG pathway enrichment analysis was conducted in DAVID [23] (Benjamini < 0.1).

## Competing interests

The authors declare that they have no competing interests.

## Authors' contributions

BC, MS and AJB conceived the study. BC performed the analysis and wrote the manuscript. BC, MS, HFM, DH and AJB discussed the results. All authors contributed to writing, reviewing, editing and approving the manuscript.

## Supplementary Material

Additional file 1 **Lists of differentially expressed genes**. Table S1: genes up regulated in HCC tumors comparing to HCC cell lines. Table S2: genes down regulated in HCC tumors compared to HCC cell lines. Table S3: genes differentially expressed in tumors compared to non-tumors. Table S4: genes up regulated in tumors compared to cell lines and dysregulated in tumors compared to non-tumors. Table S5: genes down regulated in tumors compared to cell lines and dysregulated in tumors compared to non-tumors. Click here for file
